# Utility of the Tourniquet Test and the White Blood Cell Count to Differentiate Dengue among Acute Febrile Illnesses in the Emergency Room

**DOI:** 10.1371/journal.pntd.0001400

**Published:** 2011-12-06

**Authors:** Christopher J. Gregory, Olga D. Lorenzi, Lisandra Colón, Arleene Sepúlveda García, Luis M. Santiago, Ramón Cruz Rivera, Liv Jossette Cuyar Bermúdez, Fernando Ortiz Báez, Delanor Vázquez Aponte, Kay M. Tomashek, Jorge Gutierrez, Luisa Alvarado

**Affiliations:** 1 Dengue Branch, Division of Vector-Borne Diseases, Centers For Disease Control and Prevention, San Juan, Puerto Rico; 2 Ponce University School of Medicine, Ponce, Puerto Rico; University of North Carolina at Chapel Hill, United States of America

## Abstract

Dengue often presents with non-specific clinical signs, and given the current paucity of accurate, rapid diagnostic laboratory tests, identifying easily obtainable bedside markers of dengue remains a priority. Previous studies in febrile Asian children have suggested that the combination of a positive tourniquet test (TT) and leucopenia can distinguish dengue from other febrile illnesses, but little data exists on the usefulness of these tests in adults or in the Americas. We evaluated the diagnostic accuracy of the TT and leucopenia (white blood cell count <5000/mm^3^) in identifying dengue as part of an acute febrile illness (AFI) surveillance study conducted in the Emergency Department of Saint Luke's Hospital in Ponce, Puerto Rico. From September to December 2009, 284 patients presenting to the ED with fever for 2–7 days and no identified source were enrolled. Participants were tested for influenza, dengue, leptospirosis and enteroviruses. Thirty-three (12%) patients were confirmed as having dengue; 2 had dengue co-infection with influenza and leptospirosis, respectively. An infectious etiology was determined for 141 others (136 influenza, 3 enterovirus, 2 urinary tract infections), and 110 patients had no infectious etiology identified. Fifty-two percent of laboratory-positive dengue cases had a positive TT versus 18% of patients without dengue (P<0.001), 87% of dengue cases compared to 28% of non-dengue cases had leucopenia (P<0.001). The presence of either a positive TT or leucopenia correctly identified 94% of dengue patients. The specificity and positive predictive values of these tests was significantly higher in the subset of patients without pandemic influenza A H1N1, suggesting improved discriminatory performance of these tests in the absence of concurrent dengue and influenza outbreaks. However, even during simultaneous AFI outbreaks, the absence of leucopenia combined with a negative tourniquet test may be useful to rule out dengue.

## Author Summary

In the Americas, the incidence and severity of dengue cases has increased dramatically in the past 30 years. Early diagnosis and initiation of appropriate therapy can substantially reduce dengue morbidity and mortality. However the absence of a point-of-care diagnostic test and the non-specific clinical signs and symptoms in early disease make differentiating dengue from other acute febrile illnesses challenging. Identifying dengue during an outbreak of another disease is especially difficult. The combination of a simple bedside test, the tourniquet test (TT), and a readily available laboratory test, the white blood cell count, has been reported to be a useful triage tool for identifying children with dengue in Asia, but little information exists on the performance of these tests in the Americas or among adults. We evaluated the utility of these tests in the setting of a concurrent influenza epidemic in Puerto Rico in 2009. A positive TT or leucopenia (white blood cell count <5000) was present in 94% of patients with laboratory proven dengue. Patients without either of these findings rarely had dengue. Our study indicates that a combination of two rapid, widely available tests can assist clinicians in distinguishing dengue from other illnesses with similar signs and symptoms.

## Introduction

Dengue, the disease caused by four related but distinct dengue viruses (DENV), is now considered the most important arthropod-borne disease worldwide. It is transmitted through the bite of an infected mosquito, usually *Aedes aegypti* or *Aedes albopictus* [1], and is endemic to tropical and subtropical regions. Dengue affects 50–100 million people each year. In 2007, more than 890,000 dengue cases were reported in the Americas. Globally, 500,000 patients with dengue, mostly children, require hospitalization and at least 12,500 die each year [2]. There is significant year-to-year variation in the incidence of dengue, with large outbreaks typically occurring in 3- to 5-year cycles.

Island-wide epidemics of dengue have been identified in Puerto Rico since 1915 [3] but similar to other countries in the Western Hemisphere, dengue epidemics have increased in frequency and severity over the past 20 years [4]. During the last large island-wide dengue outbreak in 2007, disease appeared to be more severe than in previous large outbreaks, with more hospitalizations and cases of dengue hemorrhage fever (DHF), and a high proportion of cases with hemorrhagic manifestations [5]. In 2007, a total of 10,508 suspected dengue cases were reported to the passive dengue surveillance system (PDSS) in Puerto Rico; 53% were hospitalized, 32% had hemorrhage and less than 1% died. One lesson learned from this outbreak was that most of the laboratory-confirmed fatal cases had a delay in diagnosis and treatment initiation, highlighting the importance of timely dengue diagnosis [5].

While morbidity and mortality has been linked to delayed provision of supportive treatment [6], case fatality rates for severe dengue infections, including DHF and dengue shock syndrome (DSS), can be reduced from 10% to 20% to less than 1% with early diagnosis and proper treatment [7]–[8]. However, there is no rapid, point-of-care diagnostic test available, and the clinical diagnosis of dengue may be challenging, as it usually presents with non-specific symptoms, including fever, headache and myalgia. Therefore, dengue is difficult to distinguish from other AFIs, such as influenza, leptospirosis, and enteroviral infections. In 2007, a simultaneous outbreak of acute gastroenteritis and dengue at the height of the dengue outbreak made recognition of dengue difficult, especially among those with warning signs for severe dengue such as persistent vomiting and abdominal pain. The concurrent outbreaks threatened to overwhelm the emergency and inpatient capacity of many Puerto Rican hospitals. In addition, simultaneous outbreaks of dengue and leptospirosis [9], dengue and measles [10], and dengue and influenza [11] in Puerto Rico caused similar difficulties with the clinical recognition, diagnosis, and management of dengue in the past.

The tourniquet test (TT) has been recommended as a tool to differentiate dengue from other AFIs [12]. Studies from Thailand found that a positive TT in combination with leucopenia could distinguish dengue from other AFI in children [13]–[15]. The aim of our study was to evaluate the accuracy and usefulness of the TT and leucopenia (white blood cell count <5,000/mm^3^) in identifying dengue among patients with AFI in a setting with a large number of adult cases and during concurrent dengue and influenza outbreaks.

## Materials and Methods

### Ethics statement

The study design and consent process was approved by the Institutional Review Boards (IRB) at the Centers for Disease Control and Prevention (CDC protocol # 5638) and Ponce University School of Medicine. Written consent was not required by the IRB as data was analyzed anonymously, the project was considered enhanced public health surveillance and the risk to the patient was considered minimal to none. Data was stripped of personal identifiers for analysis purposes and the database did not include any information that could link back to individual patients. Dengue is reportable by law in Puerto Rico and this project used the same reporting form used in the PDSS and no additional blood specimens were obtained in this project beyond what are normally collected for routine surveillance. Verbal consent was obtained for performing nasopharyngeal testing for influenza and for conducting the tourniquet test, a standard non-invasive dengue diagnostic test. A patient information sheet in Spanish written at an elementary-school level explaining the purpose of the study and the rationale and nature of these tests was read to and given to the patient. Verbal consent for performance of the influenza testing was documented by recording the results of the rapid influenza test on a separate sheet. The bottom half of the sheet, with explanation of test results in Spanish, was given to the patient and the top half was kept in a locked cabinet inside a secure CDC facility with access available only to study investigators.

### Study population

The PDSS in Puerto Rico has been in operation for more than 30 years as previously described [5]. In 2009, the PDSS at the Saint Luke's Episcopal Hospital, in Ponce, Puerto Rico, was augmented to include surveillance for all AFI. Children and adults presenting to the emergency department (ED) were eligible for enrollment if they met the following case definition of AFI: documented fever of ≥38.0°C at presentation to the ED or history of fever that persists for 2 to 7 days without identified source of fever. Those with an identifiable source of fever, including but not limited to diagnoses of otitis media, sinusitis, pneumonia, cellulitis, impetigo, wound infection, pyelonephritis, osteomyelitis or varicella were excluded. Saint Luke's Episcopal Hospital is a 425 bed tertiary care hospital that serves more than 54,000 patients in their ED each year. The Saint Luke's Episcopal Hospital is one of the largest hospitals outside of the San Juan metropolitan area and serves as the primary referral hospital for a population of more than 500,000 people in southern Puerto Rico [16].

### Study protocol

The surveillance period was from September 29, 2009 through December 18, 2009. All patients presenting for medical care at the ED of Saint Luke's Episcopal Hospital who met the case definition above were enrolled in the surveillance system. At the time of enrollment, study personnel explained the purpose of the surveillance project and obtained verbal consent for participation. Participants were interviewed to collect demographic data included on the standard PDSS data collection form, including age, sex, place of residence, and days from symptom onset to specimen collection. Clinical data that were recorded included the duration of fever, the presence or absence of headache, eye pain, myalgia, arthralgia, rash, nausea and vomiting, and hemorrhagic manifestations, including positive TT. Laboratory data recorded on the form include white blood cell count, platelet count, and highest and lowest hematocrit values. Performance of a white blood cell count, blood and urine cultures and other laboratory tests were at the discretion of the attending physician in the course of routine patient care but were not part of the study protocol. For all patients, a blood sample and two nasopharyngeal samples were collected for diagnostic testing.

A TT was performed by trained study personnel. The standard TT was performed by inflating a blood pressure (BP) cuff on the upper arm of the patient to a point mid way between their systolic and diastolic pressure for 5 minutes, and then counting the number of petechiae in a 2.5 cm^2^ area on the volar aspect of the forearm just distal to the antecubital fossa 2 minutes after releasing the BP cuff. The TT was considered positive if 10 or more petechiae were identified. Leucopenia was defined as a total white blood cell count of <5,000/mm^3^, in keeping with previously published criteria from Asia and the Americas [15], [17].

### Laboratory testing

Two nasopharyngeal samples were obtained. The first sample was tested onsite by using the QuikVue Influenza A + B rapid influenza test (Quidel Corporation, San Diego, CA), and the second sample placed in viral transport media and refrigerated until transport to the CDC's Dengue Branch for confirmatory influenza testing by polymerase chain reaction (PCR) testing. A 5–10 ml venous blood sample was collected, immediately refrigerated at 4°C, centrifuged on site, and transported on ice within 3 days to the Dengue Branch for further testing. Samples were initially tested for the presence of DENV via serotype-specific reverse-transcriptase PCR (RT-PCR) [18]–[19], DENV-specific non-structural protein-1 assay (NS-1) [20], and an anti-DENV IgM enzyme-linked immunosorbent assay (MAC-ELISA) [21]. Samples with sufficient quantity of serum remaining were subsequently transported on ice to the Bacterial Zoonosis Branch of the CDC in Atlanta for testing for leptospirosis. Specimens were screened for IgM antibodies to leptospirosis by using the rapid dipstick ELISA ImmunoDOT kit (GenBio, Inc., San Diego, CA). Specimens with positive or borderline results with the ImmunoDOT kit were further tested by using the microscopic agglutination test (MAT) [22]. Patient sera were serially diluted in the MAT and mixed with a panel of 20 *Leptospira* reference antigens that represented 17 serogroups. Resulting agglutination titers were read by using darkfield microscopy, and the final titer was expressed as the reciprocal of the last well that agglutinates 50% of the antigen. Samples from patients with illness of <3 days duration and with sufficient sera remaining were shipped at −70°C to the Picornavirus Laboratory at CDC and tested for enteroviruses by a pan-enterovirus real-time RT-PCR by using primers and probe targeting the 5′ non-translated region [23]. In PCR-positive specimens, the enterovirus type was identified by semi-nested PCR amplification and sequencing of a portion of the region encoding the VP1 capsid protein, as previously described [24]. All personnel performing laboratory testing were unaware of the clinical condition of the patient including TT and WBC count results.

### Analyses

Data was initially entered into a Microsoft Access database (Access 2007; Redmond WA) and exported to SAS version 9.2 (SAS Institute, Cary, NC). We calculated the sensitivity, specificity, positive predictive value (PPV), and negative predictive value (NPV) of leucopenia and a positive TT, both in isolation and in combination, to differentiate dengue from other AFI. Proportions were compared using 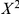 test or Fisher's exact test as appropriate. For each test, statistical significance was considered to be a P value≤0.05.

## Results

A total of 284 patients with AFI were enrolled. Thirty-one (11%) patients were laboratory confirmed as having dengue, 136 (48%) influenza and 3 (1%) were diagnosed with enterovirus. Dual infections were confirmed in two additional patients; one patient had influenza and dengue and the other had influenza and leptospirosis. These two patients were excluded from data analysis. Urinary tract infections were found in two patients (*Escherichia coli* and *Staphlococcus saprophyticus*). None of the patients had a positive blood culture. No etiology was identified among the remaining 110 enrolled patients. Serotype information was available by PCR from 20/31 laboratory-confirmed pure dengue infections, 18 were DENV-4 and 1 each were DENV-1 and DENV-2.

A TT was performed on 247 (88%) patients, of whom 54 (22%) had a positive result (Table 1). No patient had an adverse event from performance of the TT or was unable to tolerate the procedure. Half (52%) of the patients with laboratory-confirmed dengue had a positive TT while 18% of patients with influenza and 17% of patients with other AFI had a positive TT. WBC results were available for 276 (98%) patients. Dengue patients were also significantly more likely to have leucopenia (87%) than influenza patients (44%, *P*<0.001) and patients with other AFI (12%, *P*<0.001). Forty five percent of dengue patients had a positive TT and leucopenia, whereas <10% of patients with either influenza (7%, P<0.0001) or another AFI (5%, P<0.0001) had both a positive TT and leucopenia. Almost all (94%) dengue patients had either a positive TT or leucopenia compared with 57% of influenza patients and 26% of patients with other AFI.

**Table 1:** Tourniquet test and leucopenia results among patients with dengue, influenza or other AFI.

Diagnosis	TT					Leucopenia*					TT and Leucopenia					TT or Leucopenia				
	Pos	Neg	% Pos	95% CI†	PV**	Pos	Neg	% Pos	95% CI	PV	Pos	Neg	% Pos	95% CI	PV	Pos	Neg	% Pos	95% CI	PV
**Dengue(n = 31)**	16	15	52	0.35–0.68	—	27	4	87	0.71–0.95	—	14	17	45	0.29–0.62	—	29	2	94	0.79–0.98	–
**Influenza(n = 136)**	21	97	18	0.12–0.26	0.001	56	76	44	0.34–0.51	0.001	8	107	7	0.04–0.13	0.0001	69	53	57	0.48–0.65	0.0001
**Other AFI** ¶ **(n = 115)**	17	81	17	0.11–0.27	0.002	13	100	12	0.07–0.19	0.001	5	92	5	0.02–0.12	0.0001	25	73	26	0.18–0.35	0.0001

TT = tourniquet test; Pos = positive; Neg = negative; CI = confidence interval.

*Leucopenia defined as a white cell count of <5,000.

**PV = P values for difference in proportion between dengue and influenza patients and dengue and other AFI patients using X^2^ and Fisher's exact test.

**†:** Calculated via Wilson Score Interval method.

**¶:** Other AFI does not include patients with dengue or influenza.

When compared by age group, a positive TT was more common among laboratory-confirmed dengue patients aged ≤15 years than dengue patients >15 years old (67% vs. 46%), but this difference was not statistically significant (P = 0.43) (Table 2). Among patients aged ≤15 years, dengue patients were more likely to have a positive TT (67%) than patients with influenza (18%) or other AFI (17%). However, dengue patients were not more likely to have leucopenia when compared with influenza patients in this age group (67% vs. 54%, respectively, P = 0.98). Among patients >15 years old, a higher proportion of patients with dengue had leucopenia (96%) when compared with influenza patients (33%) and patients with other AFI (11%) (P<0.001). The proportion of dengue patients who presented with both a positive TT and leucopenia was similar for both age groups (44% vs. 46%). A higher proportion of dengue patients were positive for both tests either alone or in combination when compared with patients with influenza and patients with other AFI regardless of the patient's age. However, the difference was only statistically significant among patients in the older age group.

**Table 2:** Tourniquet test and leucopenia as diagnostics markers among patients by age group.

	Tourniquet Test (TT)				Leucopenia(<5000 wbc/mm^3^)				TT and leucopenia				TT or leucopenia			
Diagnosis	Pos	Neg	% Pos	95% CI*	Pos	Neg	% Pos	95% CI*	Pos	Neg	% Pos	95% CI*	Pos	Neg	% Pos	95% CI*
**Age≤15 years**																
Dengue	6	3	67	0.35–0.88	6	3	67	0.35–0.88	4	5	44	0.19–0.73	8	1	89	0.57–0.98
Influenza	9	41	18	0.10–0.31	29	25	54	0.41–0.66	4	45	8	0.03–0.19	34	19	64	0.51–0.76
Other AFI†	8	38	17	0.09–0.31	7	51	12	0.06–0.23	2	44	4	0.01–0.15	13	33	28	0.17–0.43
**Age>15 years**																
Dengue	10	12	46	0.27–0.65	21	1	96	0.78–0.99	10	12	46	0.26–0.65	21	1	96	0.78–0.99
Influenza	12	56	18	0.10–0.28	27	51	33	0.24–0.44	4	62	6	0.02–0.15	35	34	51	0.39–0.62
Other AFI†	9	43	18	0.10–0.30	6	49	11	0.05–0.21	3	47	6	0.02–0.16	12	40	23	0.14–0.37

TT = tourniquet test; AFI = acute febrile illness; CI = confidence interval; Pos = positive; Neg = negative, wbc = white blood cells, mm^3^ = cubic milliliter.

*Calculated via Wilson Score Interval method.

**†:** Other AFI does not include patients with dengue or influenza.

The TT used alone correctly identified half (sensitivity 52%) of the patients who had dengue (Table 3). The TT performed better in identifying patients who did not have dengue (specificity 82%), and a negative TT was highly associated with the absence of disease (NPV 92%). Neither the specificity nor the NPV of the TT changed significantly when influenza patients were excluded. In contrast, the presence of leucopenia alone identified most laboratory-confirmed dengue cases (sensitivity 87%). However, the specificity of leucopenia was less than that of the TT (specificity 72% vs. 82%, respectively), and it was highly affected by the presence of influenza patients. The combination of a negative TT and normal white cell count correctly identified most patients who did not have dengue (94% specificity), whereas having either a positive TT or leucopenia correctly identified a similar proportion of patients with dengue (94% sensitivity).

**Table 3:** Sensitivity, specificity, and predictive values of tourniquet test and leucopenia for laboratory-confirmed dengue.

	TourniquetTest (TT)(95% CI)	Leucopenia(95% CI)	TT & Leucopenia(95% CI)	TT or Leucopenia(95% CI)
**All patients (n = 282)** *				
Sensitivity	51.6 (33–69)	87.1 (69–96)	45.2 (28–64)	93.6 (77–99)
Specificity	82.4 (76–87)	71.8 (66–77)	93.9 (89–96)	57.2 (50–64)
PPV	29.6 (18–43)	28.1 (19–38)	51.9 (31–69)	23.6 (17–32)
NPV	92.2 (87–95)	97.8 (94–99)	92.1 (88–95)	98.4 (94–99)
**All patientsexceptinfluenzapatients(n = 146)** †				
Sensitivity	51.6 (33–69)	87.1 (69–96)	45.2 (28–64)	93.6 (77–99)
Specificity	82.7 (73–89)	88.5 (81–93)	94.9 (88–98)	73.7 (64–82)
PPV	48.5 (31–66)	67.5 (51–81)	73.7 (49–90)	53.7 (39–66)
NPV	84.4 (75–91)	96.2 (90–99)	84.4 (76–90)	97.2 (90–99)

TT = tourniquet test; PPV = Positive Predictive Value; NPV = Negative Predictive Value.

*Data available on TT results for 247 patients and for WBC count for 276 patients.

**†:** Data available on TT results for 129 patients and for WBC count for 144 patients.

The relationship between TT results and platelet count, a marker of disease severity in dengue, is shown in Table 4. A positive TT was associated with laboratory-confirmed dengue in patients with a platelet count greater than 100,000 (P = 0.0001) but not in patients with a platelet count below this level. For patients without dengue, no association was seen between the platelet level and a positive TT.

**Table 4:** Percent of positive tourniquet tests for lab-confirmed dengue versus non–dengue patients by platelet count.

Diagnosis	Platelets≤100K			Platelets>100K		
	No.	%	*PV* *	No.	%	*PV* *
Dengue	9/22	40.9	0.672	7/9	77.8	0.0001
Non-dengue	2/8	25.0		35/205	17.5	

*PV = P values for difference in proportion between dengue and non-dengue patients using X^2^ and Fisher's exact test.

## Discussion

Our study sought to evaluate the usefulness of a positive TT and leucopenia alone and in combination in identifying patients with dengue among children and adults with AFI in an ED of a dengue endemic area. Previous studies examining the use of one or both of these tests have primarily been performed in Asia among children suspected to have dengue and who required hospital admission [13]–[15], [25]–[27]. While direct comparisons with the results of Asian studies are difficult because of differences in disease epidemiology and study design, our study supports the findings of previous studies conducted in Thailand [14], [15], which found that the presence of either leucopenia, a positive TT, or both is helpful in distinguishing between patients with and without dengue. In our study and others [17], [28], [29], leucopenia alone was found to be an especially good indicator of dengue among adults. Future studies examining the predictive value of the tourniquet test for the diagnosis of dengue in adults should evaluate a positive TT in combination with leucopenia.

As described in previous studies, we found that a positive TT alone was specific but not sensitive in distinguishing dengue from other AFI [25], [27]–[29]. This is especially true when the WHO cut-off of 20 or more petechiae per 2.5 cm^2^ is used. As in other studies, we chose to maximize detection of positive dengue cases (or the sensitivity of the tourniquet test) by using the cut-off of 10 or more petechiae per 2.5 cm^2^ [13], [14], [30]. The presence of either a positive TT or leucopenia correctly identified 94% of patients who had dengue, and the absence of a positive TT or leucopenia was highly predictive of the absence of disease with a NPV >98%. That is, less than 2% of enrolled patients with neither a positive TT nor leucopenia had dengue.

Compared with the data reported from Thailand on the combined performance of the TT and leucopenia in identifying dengue patients, our results showed a lower sensitivity (45% vs.74%) and PPV (52% vs.73%–83%) but similar specificity (94% vs. 86%) [14]–[15]. Our finding of lower sensitivity most likely reflects differences in study design. These previous studies primarily enrolled hospitalized patients who then received daily TTs until the day of defervescence. Our study participants had a single TT performed at initial presentation to the ED. Previous research has demonstrated that the sensitivity of the TT depends on repeated testing and the timing of the test with respect to the day of illness with sensitivity increasing as a patient nears defervescence [14], [26]. Most (68%) of our participants were enrolled within three days of symptom onset so that the sensitivity of the TT found in our study corresponds to the day −4/day −3 values found in the study from Thailand (52% versus 46%–56%) [14]. We feel that using values solely from the time of initial patient contact is more useful, as it uses only information that is available to physicians at the time of initial triage.

Given that the study was performed in the setting of a concomitant influenza pandemic (CDC, unpublished data) and we did not restrict enrollment to suspected dengue cases that required hospitalization, our study also provides data on the performance of these triage criteria for dengue during periods of high influenza transmission. The lower PPV in our study largely reflects the effect of the study being conducted among patients with AFI (versus only those suspected of having dengue) at the time of a major outbreak of another AFI. While the specificity and PPV of the combination of tests was lower when cases with pandemic influenza A H1N1 were included, overall performance was still good. Reanalyzing the data after excluding influenza cases resulted in a PPV for the combination of leucopenia and positive TT of 74%, similar to previously published estimates [14], [15]. As only 12% of patients without dengue or influenza had leucopenia in our study, there was only a minor decrease in the NPV upon excluding influenza patients for the combination of leucopenia and positive TT (92% vs 84%).

Our study has some important limitations. It was performed at an ED in a tertiary-level referral hospital with a large catchment area, and patients seeking care at this facility are more likely to have severe disease than patients who seek care at lower-level facilities. Thus, dengue cases in our study were likely not representative of the whole spectrum of dengue occurring in Puerto Rico. Data were missing on TT and white blood cell count results for approximately 12% and 2% percent of patients, respectively. The 35 participants who did not have a TT performed did not differ significantly from those who did have a TT performed in terms of sex or median age. No cases of dengue were diagnosed among the patients who did not have a TT performed. In most cases, only one serum specimen was available for dengue testing, leading to the possibility that some true dengue cases were not detected and misclassified as dengue negative. A comprehensive testing strategy, including a highly sensitive, single-plex anti-DENV RT-PCR, a NS-1 assay, and a MAC ELISA, was used to try to limit the amount of this misclassification bias.

The effects of dengue serotype and immunological status (primary versus secondary dengue infection) on the incidence of a positive TT or leucopenia have not yet been sufficiently investigated. Few studies have examined the role of these variables on the performance of these tests and these studies have usually been relatively small. Even fewer studies have had an adequate number of cases with both virological confirmation and immunological data to look at both variables simultaneously and stratify serotype specific results by immunological status, an important potential confounder. A positive TT was more frequently seen in children with dengue in Nicaragua when DENV-1 was the predominant serotype compared to an era when DENV-2 was circulating widely [31]. However this difference was not evident in the sub-group of virologically confirmed cases and the study did not stratify between primary and secondary infections. No difference in white blood cell count was seen between 385 adults with DENV-2 or DENV-3 infection in Taiwan [32], but significantly more DENV-3 patients had secondary infections making accurate comparisons between groups difficult. No differences were seen in the proportion of European travelers with a positive TT between patients with a primary or secondary immune response [33], but the small number of patients with a secondary immune response limited the power of the study. No significant differences for TT positivity or leukocyte count in primary and secondary infections were seen among 89 hospitalized DENV-1 patients in Fiji [34].

Overall our study indicates that a combination of two rapid, widely available tests can assist clinicians in distinguishing dengue from other AFIs that have similar clinical signs and symptoms. Previous investigators have reported that the combination of the TT and leucopenia is more accurate in identifying patients with dengue than the World Health Organization's 1997 clinical case definition [15], and our data supports the contention that few patients with dengue are likely to be missed when these criteria are used. Twenty-nine of the 31 laboratory-positive dengue patients had either a positive TT or leucopenia. The two dengue patients that were not detected by these two tests were thrombocytopenic.

Our study and others [31] suggest that the TT may be useful in identifying dengue patients before a major decrease in platelet count, a group for whom dengue is often overlooked as a diagnostic possibility in Puerto Rico. Patients in our study population with AFI who had a negative TT and normal WBC appear to be at low risk of having dengue, and patients with both leucopenia and a positive TT have a high likelihood of having dengue, even in the setting of an outbreak of another clinically similar illness. Increased emphasis should be placed on determining the usefulness of the TT in combination with white blood cell count in identifying patients with dengue in Puerto Rico and elsewhere in the Americas. Further exploration of the sensitivity, specificity and predictive value of these tests by day of illness would be of particular benefit for clinical decision making. Additional larger studies should also be conducted to explore the effect of dengue serotype and immune status on the diagnostic performance of these tests.

## Supporting Information

Flowchart S1 **STARD Flowchart for tourniquet test.**
(PDF)

Flowchart S2 **STARD Flowchart for white blood cell count<5000/mm.**
(PDF)
