# Supplementary figures and images for: Utility of the Tourniquet Test and the White Blood Cell Count to Differentiate Dengue among Acute Febrile Illnesses in the Emergency Room
Source: PLoS Negl Trop Dis. 2011 Dec 6;5(12):e1400. doi: 10.1371/journal.pntd.0001400 (PMC3232191; doi:10.1371/journal.pntd.0001400)

STARD Flowchart for  
tourniquet test

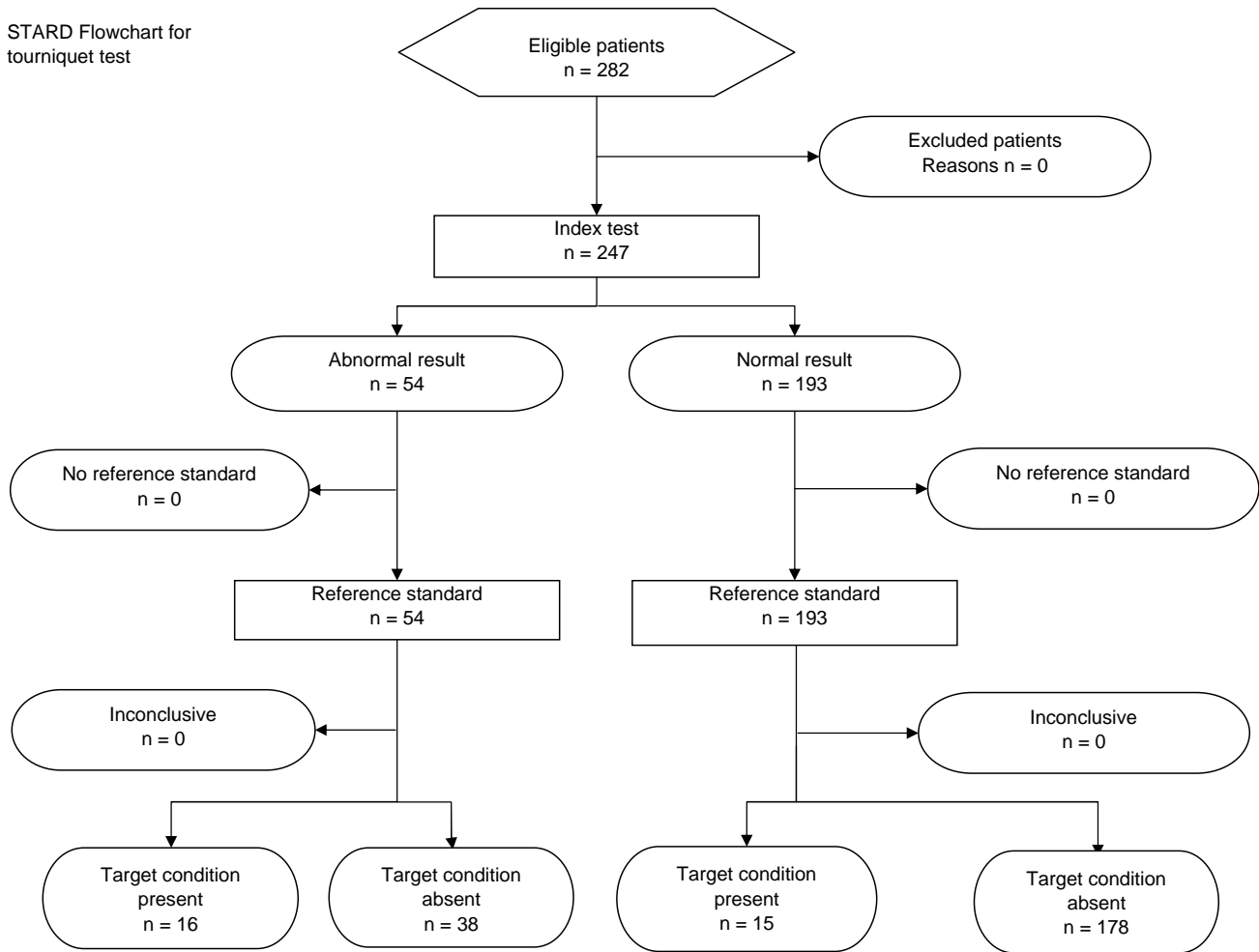

Supplement: Flowchart S1 — STARD Flowchart for tourniquet test. (PDF) [file pntd.0001400.s001.pdf]

STARD Flowchart for  
white blood cell count < 5000/mm

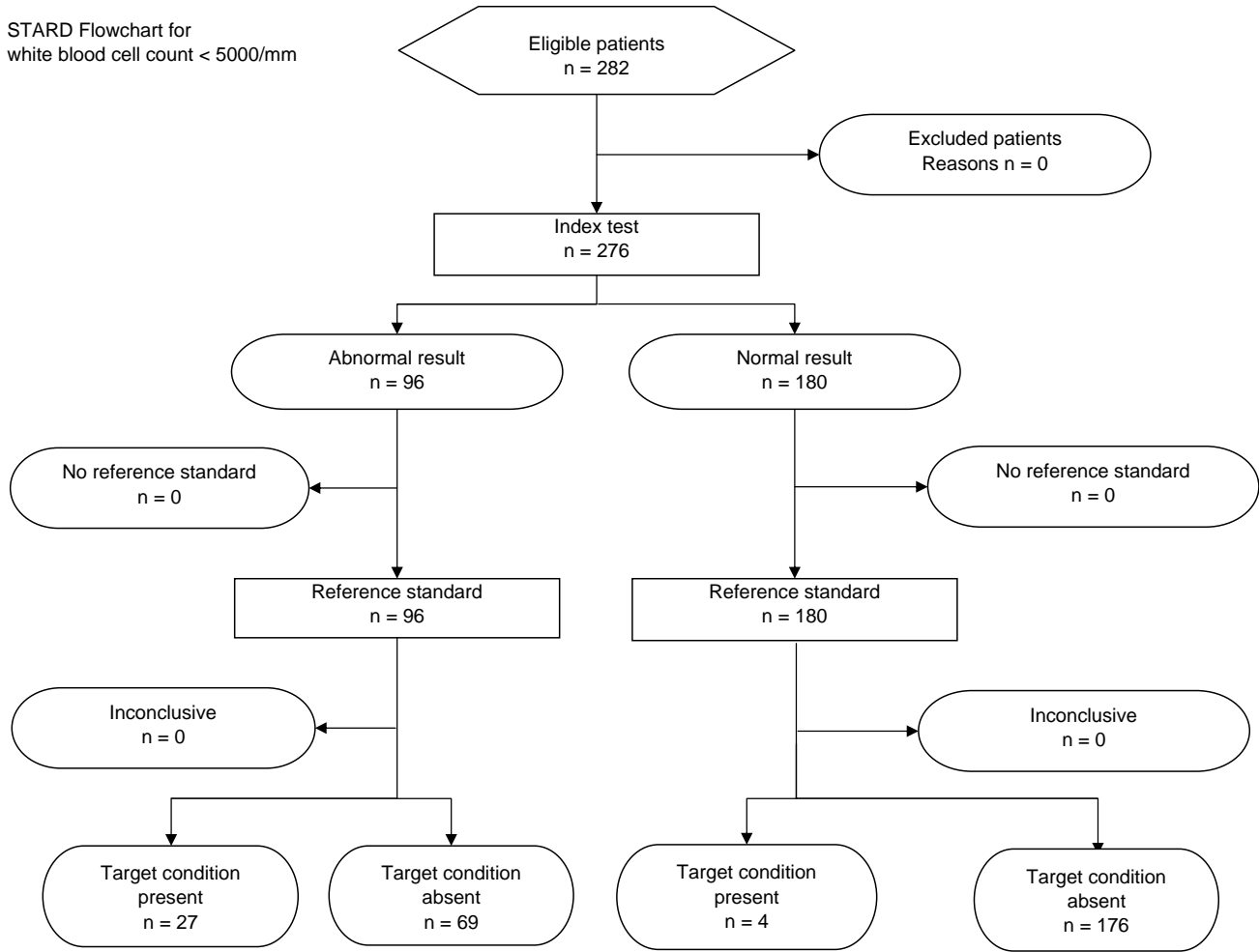

Supplement: Flowchart S2 — STARD Flowchart for white blood cell count<5000/mm. (PDF) [file pntd.0001400.s002.pdf]
